# RESPONDERS AND NONRESPONDERS WITH KNEE OSTEOARTHRITIS TO TRANSCRANIAL DIRECT CURRENT STIMULATION

**DOI:** 10.1093/geroni/igae098.0811

**Published:** 2024-12-31

**Authors:** Juyoung Park, Heling Tong, Yixin Kang, Hongyu Miao, Hyochol Ahn

**Affiliations:** University of Arizona, Tucson, Arizona, United States; Florida State University, Tallahassee, Florida, United States; Florida State University, Tallahassee, Florida, United States; Florida State University, Tallahassee, Florida, United States; University of Arizona, Tucson, Arizona, United States

## Abstract

Knee osteoarthritis (KOA), a common musculoskeletal condition in older adults, often leads to long-term disability. Transcranial direct current stimulation (tDCS) has received much attention as a noninvasive treatment for knee pain due to its neuromodulatory effect. However, there is variability in the response to tDCS among KOA patients, and little is known about the factors predicting response to tDCS for knee pain. The purpose of the study was to identify responders and nonresponders to the tDCS intervention and characterize their differences in knee pain reduction post intervention. Sixty participants assigned to the home-based active tDCS (five 20-minute sessions of 2 mA direct current), under real-time supervision by the research staff, were included; 41 were classified as responders (64.90 years, 41.50% White, 61.00% women) and 19 were classified as nonresponders (66.21 years, 47.40% White, 78.90% women). Changes in pain intensity scores from baseline to post intervention indicated more significant decreases in responders than in nonresponders (p <.001); the mean decrease in pain intensity from baseline to post intervention was 34.17 (SD = 17.40) for the responder group and 2.26 (SD = 10.70) for the nonresponder group. The responder group showed higher BMI, height, and weight than the nonresponder group. The results indicated that responder and nonresponder groups and their treatment outcomes can be predicted for tailoring tDCS interventions. More research is needed to identify reasons for nonresponse to tDCS (e.g., medical comorbidities) to reduce nonresponse rates to tDCS.

